# Prevalence and risk factors for hyperuricemia and hyperuricosuria in patients with hematologic malignancies

**DOI:** 10.3389/fmed.2024.1343000

**Published:** 2024-05-30

**Authors:** Thanaput Kunlayawutipong, Thanawat Rattanathammethee, Teerachat Punnachet, Nonthakorn Hantrakun, Pokpong Piriyakhuntorn, Sasinee Hantrakool, Chatree Chai-Adisaksopha, Ekarat Rattarittamrong, Adisak Tantiworawit, Lalita Norasetthada, Worawit Louthrenoo

**Affiliations:** ^1^Department of Internal Medicine, Faculty of Medicine, Chiang Mai University, Chiang Mai, Thailand; ^2^Division of Hematology, Department of Internal Medicine, Faculty of Medicine, Chiang Mai University, Chiang Mai, Thailand; ^3^Division of Rheumatology, Department of Internal Medicine, Faculty of Medicine, Chiang Mai University, Chiang Mai, Thailand

**Keywords:** hyperuricemia, hyperuricosuria, myeloproliferative neoplasms, lymphoma, risk factors

## Abstract

**Introduction:**

Hyperuricemia is a common complication of hematologic malignancies, and hyperuricosuria in this population has shown conflicting results. This study aimed to determine the prevalence of hyperuricemia and parameters associated with serum uric acid (SUA) and urine uric acid (UUA) in patients with lymphoma and myeloproliferative neoplasms (MPN).

**Methods:**

This cross-sectional study included adult patients with newly diagnosed lymphoma and MPN at the university-based hospital. Clinical characteristics were collected, and independent risk factors for hyperuricemia and hyperuricosuria were determined using multiple logistic regression.

**Results:**

One hundred and sixty-five patients were included with a median age of 55 years (45.5–64) and 51.5% were males. There were 91 patients (55.2%) with lymphoma and 74 cases (44.8%) of MPN. Overall, hyperuricemia was prevalent in 43.6% with a median SUA of 6.3 mg/dl (4.6–8) and hyperuricosuria was detected in 39.4% with a median 24-h UUA of 545 mg (365.4–991). Hyperuricemia was observed in patients with lymphoma and MPN in 20.9% and 71.6%, respectively, and hyperuricosuria in 15.4% and 68.9%, respectively. In lymphoma patients, estimated glomerular filtration rate (eGFR) <90 ml/min/1.73 m^2^ and serum lactate dehydrogenase (LDH) ≥ 250 U/L were associated with hyperuricemia with odds ratio (OR) 3.24, 95% confidence interval (CI) 1.95–11.07, *p* = 0.006 and OR 2.07, 95%CI 1.62–6.97, *p* = 0.039), and only elevated serum LDH was related to hyperuricosuria (OR 2.37, 95%CI 1.56–14.29, *p* = 0.036). In MPN patients, hemoglobin levels <10 g/dl and serum LDH ≥ 640 mg/dl were independent risk factors of hyperuricosuria (OR 1.88, 95%CI 1.42–8.39, *p* = 0.045 and OR 6.21, 95%CI 1.49–25.74, *p* = 0.012).

**Conclusion:**

Hyperuricemia in patients with hematologic malignancies was common, notably MPN, and parameters associated with hyperuricosuria were provided. In addition to the utilization of allopurinol in patients at high risk of tumor lysis syndrome, patients without hyperuricosuria may also be of significant interest.

## 1 Introduction

Hyperuricemia is one of the common complications of hematologic malignancies, resulting from a high proportion of cellular turnover rate. In addition, cell lysis occurs during treatment with chemotherapy or cytoreductive agents not only can aggravate elevated serum uric acid (SUA) but also increase urine uric acid (UUA) excretion and acute uric acid nephropathy is possibly anticipated. Therefore, it is generally suggested that allopurinol should be prescribed in these patients to prevent acute uric acid nephropathy (1–4). Moreover, hyperuricemia has well-described reports on the association with physical illnesses such as hypertension, impaired fasting blood glucose and type 2 diabetes, metabolic syndrome, and coronary heart disease in previous studies (5–10). These comorbidities and SUA levels should be a concern, especially in individuals with hematologic malignancies. Although the tumor lysis syndrome (TLS) is the life-threatening complication, with a prevalence ranging from 6.1% to 27.8% in patients with acute leukemia and non-Hodgkin lymphoma, that results in hyperuricemia and acute kidney injury, the study on the risk factors of hyperuricemia or hyperuricosuria remains controversial (1–4). A 5.5% incidence of *de novo* kidney stones in patients with myeloproliferative and lymphoproliferative disorders after initiation of chemotherapy had been reported (11). Uric nephrolithiasis typically develops in patients who have acidified urine, hyperuricosuria, and low urine volume. It remains to be explored whether hematologic malignancies with a high tumor cell burden contribute to a significant subset with a lesser incidence of kidney stones (12, 13).

The prevalence of hyperuricemia in hematologic malignancies has been reported to range between 18.9 and 65.5% (1, 3, 4, 14–16). Moreover, studies on UUA excretion also showed conflicting results between uric acid hyperexcretion and uric acid underexcretions (16, 17). This study aimed to determine the prevalence and risk factors of hyperuricemia and hyperuricosuria in patients with previously untreated hematologic malignancies. The study specifically examined uric acid concentration and excretion within a defined group of hematologic malignancies characterized by subacute or chronic progression and proliferative behavior. It focused on two representative diseases: myeloproliferative neoplasms (myeloid lineage) and lymphomas (lymphoid lineage). The hypothesis centered on the possibility that these distinct malignancies may exhibit divergent uric acid metabolism due to underlying differences in their pathogenic mechanisms.

## 2 Materials and methods

### 2.1 Patient selection and definitions

This cross-sectional study was conducted on adult patients with hematologic malignancies, including lymphoma (ICD-10 code: C81-C88) and myeloproliferative neoplasms (MPN) [chronic myeloid leukemia (CML) (ICD-10 code: C92.10-C92.12), polycythemia vera (PV) (ICD-10 code: D45) and essential thrombocytosis (ET) (ICD-10 code: D47.3)], between 1 July 2019 and 31 December 2022 at the Chiang Mai University Hospital, Thailand. Written informed consent was obtained from each participant before enrolling in the study. All clinical characteristics including staging, bulky status of lymphoma, serum lactate dehydrogenase (LDH) and hematological parameters were collected with de-identified patient data both during and after data collection. Inclusion criteria included treatment naïve patients with newly diagnosed lymphoma and MPN aged 18 years or above. Patients with acute leukemia were excluded from this study due to several considerations. Firstly, acute leukemia carries a high risk of tumor lysis syndrome (TLS). TLS can significantly alter uric acid metabolism, potentially confounding the interpretation of study results. Secondly, patients with acute leukemia often require prompt intervention. The emergency treatment protocols for this condition might not be compatible with the research protocol of this study, potentially compromising patient safety. Patients with pre-existing gouty arthritis, active infection, significant impaired renal function (estimated glomerular filtration rate [eGFR] < 60 ml/min/1.73 m^2^), chronic alcohol drinkers, using of over the counter drugs or herbal medicines, and users of medication that interfered with SUA levels or UUA excretion including uric-lowering drugs (e.g., allopurinol, febuxostat, probenecid benzbromarone, and sulfinpyrazone), diuretic, anti-tuberculous drugs (particularly pyrazinamide and ethambutol), high dose aspirin (more than 325 mg/day), cyclosporin, levodopa, and nicotinic acid were excluded. All demographic characteristics, types of hematologic malignancy, comorbidities, and current medication were reviewed. The laboratory investigations included completed blood count, blood chemistry, serum creatinine, SUA, urine analysis, and 24-h urine collection for UUA (24-h UUA) were performed on the same day of enrollment.

Hyperuricemia was defined as SUA level > 6.8 mg/dl (18, 19), and hyperuricosuria was defined if 24-h UUA > 700 mg on a regular unrestricted purine diet (20, 21). Adequate 24-h urine collection was confirmed in all patients with 24-h urine creatinine excretion > 15 mg/kg/day and > 20 mg/kg/day in females and males, respectively (22). The renal function was determined by the calculated eGFR using the Cockcroft-Gault formula (23). This study followed the Declaration of Helsinki and the International Conference on Harmonization Guidelines for Good Clinical Practice. The institutional ethical review board of the Faculty of Medicine, Chiang Mai University, Thailand, approved the study (study code: MED-2563-07002).

### 2.2 Sample size and statistical analysis

The prevalence of hyperuricemia in hematologic malignancies excluding acute leukemia, was considered the primary outcome of this study. For the expected prevalence of 33% of hyperuricemia in hematologic malignancies excluding acute leukemia (16), the required sample size was 165 patients for a margin of error of 5% with two-sided in estimating the prevalence with 95% confidence with 80% of study power and considering the potential loss of 8% (24). The Stata/SE software version 14.1 for Mac (StataCorp, Texas, USA) was used for statistical analysis. Results of categorical variables were expressed as proportion or percentage, and continuous variables were expressed as mean with standard deviation (SD) or median with interquartile range (IQR) depending on their distribution. The chi-square or Fisher's exact test was used to compare categorical variables, and a student's *t*-test or Mann-Whitney U test was used to compare continuous data, as appropriate. The penalized maximum likelihood logistic regression was used for small samples or rare event data. The variables were calculated as *p* < 0.1 in the univariable analysis entered the multivariable analysis. Multiple logistic regression analysis was used to identify independently associated risk factors for hyperuricemia or hyperuricosuria. Odds ratio (OR) and 95% confidence intervals (CI) were calculated for all associations. A *p* < 0.05 was considered statistically significant. All patients with missing data were excluded.

## 3 Results

**Figure 1:**
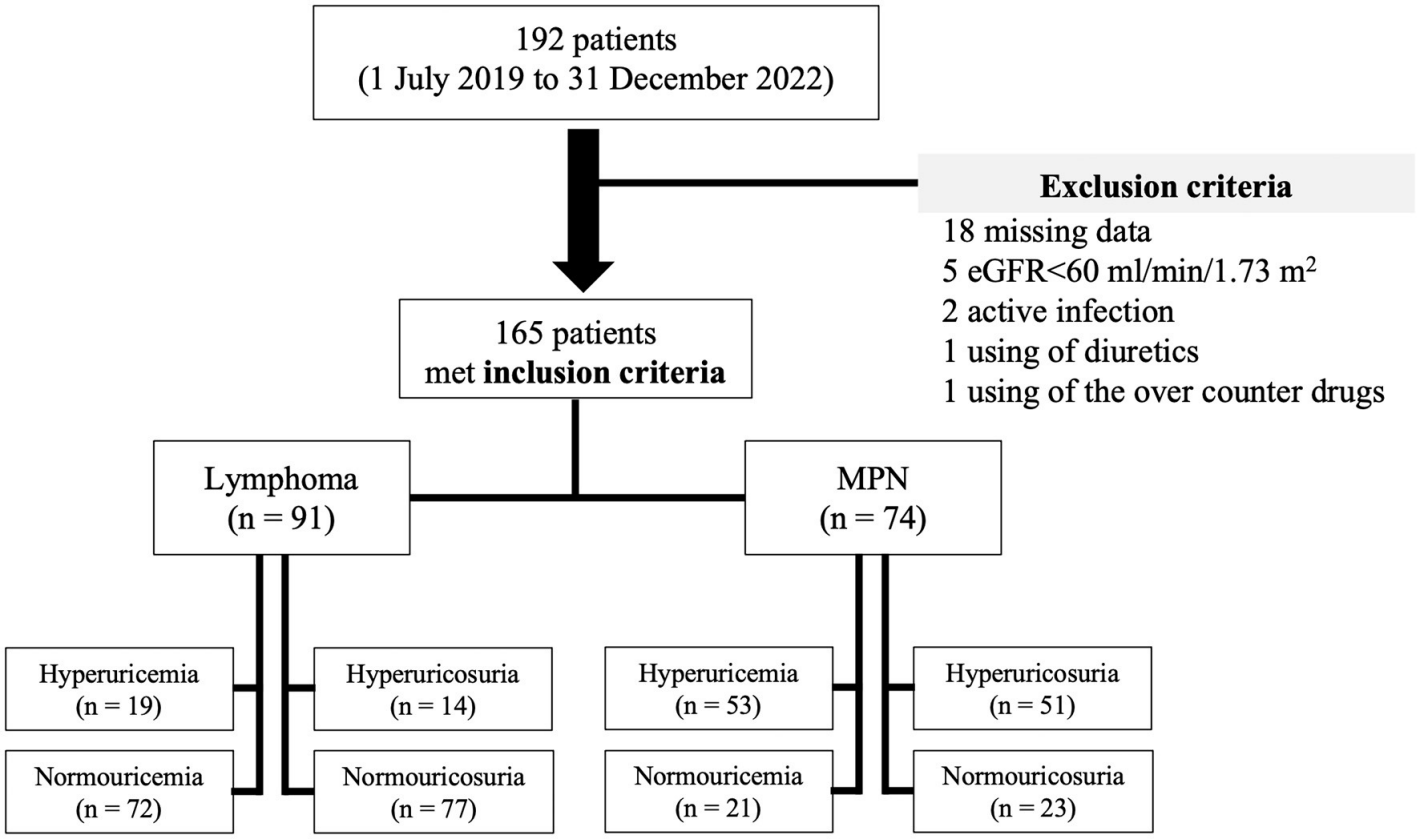
Flow diagram of total recruitment alongside inclusion and exclusion criteria.

A total 192 patients were recruited, 27 patients were excluded, and of 165 patients (51.5% male) were enrolled in the study. Median (IQR) age and body mass index (BMI) were 55 years (45.5–64) and 21.2 kg/m^2^ (19–23), respectively. Pre-existing comorbidities included diabetes mellitus in 4.8% and hypertension in 14.5%. Overall, prevalence of hyperuricemia was 43.6% (95%CI: 6.03–6.56) with median SUA of 6.3 mg/dl (4.6–8) prevalence of hyperuricosuria was 39.4% (95%CI: 518.52–596.27) with median 24-h UUA of 545 mg (365.4–991). The patients were separated by type of hematologic malignancies into lymphoma in 91 (55.2%) cases and MPN in 74 (44.8%) cases. Lymphoma patients had a median age of 60 years (51–69) with 84.6% of diffuse large B-cell lymphoma (DLBCL) and 15.4% of indolent lymphoma, while the median age of MPN patients was 48 years (39.7–58). Hypertension was slightly higher in lymphoma than in MPN patients (19.8% vs. 8.1%, *p* = 0.045). There was no difference in sex distribution, BMI, proportion of diabetes mellitus, and hemoglobin level between lymphoma and MPN patients. MPN patients (CML in 66.2%, ET in 17.6%, and PV in 16.2%) had significantly higher initial white blood cells count, platelet count, and serum LDH, while eGFR was significantly lower than patients with lymphoma. Hyperuricemia was observed in 20.9% (95%CI: 4.98–5.63) and 71.6% (95%CI: 7.59–8.26) of lymphoma and MPN patients (*p* = 0.018), respectively. Hyperuricosuria was detected in 9.9% (95%CI: 395.94–505.90) and 63.5% (95%CI: 902.1–1,013.13) of lymphoma and MPN patients (*p* = 0.005), respectively (Figure 1, Table 1).

**Table 1:** Characteristics of lymphoma and myeloproliferative neoplasm patients.

**Characteristics**	**All patients (*n* = 165)**	**Lymphoma (*n* = 91)**	**MPN(*n* = 74)**	***P*-value**
**Age**, years	55 (45.5–64)	60 (51–69)	48 (39.7–58)	< 0.001
**Male**, *n* (%)	85 (51.5)	45 (49.5)	40 (54.1)	0.639
**BMI**, kg/m^2^	21.2 (19-23)	21.1 (19–22.7)	21.5 (19-23)	0.464
**Comorbidities**, ***n*** **(%)**				
Diabetes mellitus	8 (4.8)	6 (6.6)	2 (2.7)	0.298
Hypertension	24 (14.5)	18 (19.8)	6 (8.1)	0.045
**Laboratory parameters**				
**Complete blood count**				
Hemoglobin, g/dl	10.5 (8.8–12.5)	10.6 (8.9–12.6)	10.2 (8.5–12.3)	0.628
WBC, × 10^9^/L	9.8 (6.4–77.6)	6.7 (4.6–8.7)	94.1 (18.6–263.6)	< 0.001
Platelet, × 10^9^/L	316 (222–541.2)	243 (146–326.4)	545.9 (364.5–677.7)	< 0.001
**Renal function**				
Serum creatinine, mg/dl	0.8 (0.7–1.0)	0.8 (0.6–0.9)	0.9 (0.7–1.0)	0.372
eGFR, ml/min/1.73 m^2^	81.2 (72.8–96.4)	89.0 (73.0–103.3)	79.8 (63–85)	< 0.001
Serum LDH^**#**^, U/L	477 (236.5–692.5)	254 (210–559)	643 (481–784.3)	< 0.001
**Uric profiles**				
SUA, mg/dl	6.3 (4.6–8)	5.3 (3.5–6.6)	8 (6-9)	< 0.001
Hyperuricemia^*^, *n* (%)	72 (43.6)	19 (20.9)	53 (71.6)	0.018
24-h UUA, mg	545 (365.4–991)	420 (277–561.5)	983 (612.3–1,096)	< 0.001
Hyperuricosuria^$^, *n* (%)	65 (39.4)	14 (15.4)	51 (68.9)	< 0.001

As the continuous data in this study did not follow a normal distribution, all data were reported as medians with interquartile ranges (IQR).

BMI, body mass index; MPN, myeloproliferative neoplasm; WBC, white blood cells count; eGFR, estimated glomerular filtration rate; LDH, lactate dehydrogenase; SUA, serum uric acid; 24-h UUA, 24-h urine uric acid.

^#^Normal range of serum LDH: 150–245 U/L, ^*^Serum uric acid > 6.8 mg/dl, ^$^24-h UUA > 700 mg.

Due to the difference in clinical characteristics and disease biology, the associated parameters of hyperuricemia and hyperuricosuria were separately analyzed between lymphoma and MPN patients. For lymphoma patients, the median SUA in the hyperuricemia group (*n* = 19) and in the normouricemia group (*n* = 72) was 7.9 mg/dL (7.2–8.3) and 4.6 mg/dl (3.3–5.7), respectively. DLBCL patients had a trend of slightly higher in proportion of hyperuricemia than patients with indolent lymphoma. There was no significant difference in clinical characteristics between lymphoma patients with and without hyperuricemia except that those who had hyperuricemia had a higher proportion of patients with impaired renal function (eGFR < 90 ml/min/1.73 m^2^) (73.6% vs. 44.5%, *p* = 0.035) and serum LDH ≥ 250 mg/dl (63.2% vs. 51.3%, *p* = 0.009). The proportion of patients with hyperuricosuria was also higher in patients with hyperuricemia than in the normouricemia group (57.9% vs. 4.2%, *p* < 0.001) (Table 2).

**Table 2:** Characteristics of lymphoma patients separated by hyperuricemia.

**Characteristics**	**All patients**	Hyperuricemia^*^	**Normouricemia**	***P*-value**
	**(*****n*** = **91)**	**(*****n*** = **19)**	**(*****n*** = **72)**	
**Age**, years	60 (51–69)	60 (51–67)	60.5 (50.7–70)	0.489
**Male**, *n* (%)	45 (49.5)	10 (52.7)	35 (48.7)	0.478
**BMI**, kg/m^2^	21.1 (19–22.7)	21.1 (20.1–22.5)	21 (18.7–22.7)	0.505
**Comorbidities**, ***n*** **(%)**				
Diabetes mellitus	6 (6.6)	1 (5.3)	5 (6.9)	0.633
Hypertension	18 (19.8)	3 (15.8)	15 (20.8)	0.755
**Lymphoma subtype**, ***n*** **(%)**				
DLBCL	77 (84.6)	18 (94.7)	59 (81.9)	0.285
Indolent lymphoma^§^	14 (15.4)	1 (5.3)	13 (18.1)	0.154
**Ann Arbor staging**, ***n*** **(%)**				
Stage I-II	29 (31.9)	6 (31.6)	23 (31.9)	0.322
Stage III-IV	62 (68.1)	13 (68.4)	49 (68.1)	0.425
**IPI risk group**, ***n*** **(%)**				
Low (0–1)	25 (27.5)	5 (26.3)	20 (27.7)	0.693
Low-intermediate (2)	14 (15.4)	2(10.5)	12(16.7)	0.385
High-intermediate (3)	22 (24.2)	5 (26.3)	17 (23.6)	0.271
High (4-5)	30 (32.9)	7 (36.8)	23 (31.9)	0.199
**Bulky disease**^#^, *n* (%)	20 (21.9)	5 (26.3)	15 (20.8)	0.284
**Laboratory parameters**				
**Complete blood count**				
Hemoglobin, g/dl	10.6 (8.9–12.6)	11.2 (8.1–12.3)	10.6 (8.9–12.6)	0.715
WBC, × 10^9^/L	6.7 (4.6–8.7)	7.6 (4.9–9.2)	6.7 (4.4–8.6)	0.408
Platelet, × 10^9^/L	243 (146–326.4)	266 (194–344)	238 (128–310.3)	0.183
**Renal function**				
Serum creatinine, mg/dl	0.8 (0.6–0.9)	0.9 (0.7–1.0)	0.8 (0.7–0.9)	0.420
eGFR, ml/min/1.73 m^2^	89.0 (73.0–103.3)	79 (68–95)	91.5 (76.3–104)	0.051
< 90, *n* (%)	46 (50.5)	14 (73.6)	32 (44.5)	0.035
**Serum LDH**^**@**^, U/L	254 (210–559)	545 (213–947)	252 (207–437.5)	0.060
≥250 U/L, n (%)	49 (53.8)	12 (63.2)	37 (51.3)	0.009
**Uric acid profiles**				
SUA, mg/dl	5.3 (3.5–6.6)	7.9 (7.2–8.3)	4.6 (3.3–5.7)	< 0.001
24-h UUA, mg	420 (277–561.5)	475.3 (191.2–588.6)	400.5 (287–545.5)	0.044
Hyperuricosuria^$^, *n* (%)	14 (15.4)	11 (57.9)	3 (4.2)	< 0.001

As the continuous data in this study did not follow a normal distribution, all data were reported as medians with interquartile ranges (IQR).

BMI, body mass index; DLBCL, diffuse large B-cell lymphoma; IPI, International Prognostic Index; WBC, white blood cells count; eGFR, estimated glomerular filtration rate; LDH, lactate dehydrogenase; SUA, serum uric acid; 24-h UUA, 24-h urine uric acid.

^§^Indolent lymphoma: marginal zone lymphoma (n = 9) and follicular lymphoma (n = 5), ^*^Serum uric acid > 6.8 mg/dl, ^#^Bulky disease: tumor diameter > 7.5 cm, ^@^Normal range of serum LDH: 150–245 U/L, ^$^24-h UUA > 700 mg.

In MPN patients, the median SUA in the hyperuricemia group (*n* = 53) and in the normouricemia group (*n* = 21) was 8.9 mg/dl (8, 9) and 5 mg/dl (4.6–6). Among MPN patients with hyperuricemia, the median SUA levels were 9 mg/dl (8, 9) for CML, 8 mg/dl (7.6–8.8) for ET, and 8.9 mg/dl (8, 9) for PV, with no statistically significant differences between the groups. For MPN patients with hyperuricosuria, the median 24-h UUA excretion levels were 1,069 mg (980–1,175) for CML, 1,137 mg (856–1,179) for ET, and 1,090 mg (978–1,218) for PV, with no statistically significant differences between the groups. Hyperuricosuria was more prevalent in patients with hyperuricemia compared to those with normouricemia (75.5% vs. 52.4%, *p* = 0.027) (Table 3).

**Table 3:** Characteristics of myeloproliferative neoplasm patients separated by hyperuricemia.

**Characteristics**	**All patients**	Hyperuricemia^*^	**Normouricemia**	***P*-value**
	**(*****n*** = **74)**	**(*****n*** = **53)**	**(*****n*** = **21)**	
**Age**, years	48 (39.7–58)	49 (43–58)	46 (36–57.5)	0.146
**Male sex**, *n* (%)	40 (54.1)	32 (60.4)	8 (38.1)	0.087
**BMI**, kg/m^2^	21.5 (19-23)	21.6 (19.1–23.9)	20.2 (18.4–22.5)	0.253
**Comorbidities**, ***n*** **(%)**				
Diabetes mellitus	2 (2.7)	1 (1.9)	1 (4.8)	0.507
Hypertension	6 (8.1)	5 (9.4)	1 (4.8)	0.515
**MPN subtype**, ***n*** **(%)**				
CML	49 (66.2)	35 (66.1)	14 (66.7)	0.959
ET	13 (17.6)	8 (15.1)	5 (23.8)	0.378
PV	12 (16.2)	10 (18.9)	2 (9.5)	0.335
**Laboratory parameters**				
**Complete blood count**				
Hemoglobin, g/dl	10.2 (8.5–12.3)	9.6 (8.2–11.9)	11.3 (9–12.5)	0.379
WBC, × 10^9^/L	94.1 (18.6–263.6)	83.7 (18.5–259.8)	110.9 (17.5–269.9)	0.969
Platelet, × 10^9^/L	545.9 (364.5–677.7)	522.5 (356.1–672)	635.2 (362.7–722.1)	0.389
**Renal function**				
Serum creatinine, mg/dl	0.9 (0.7–1.0)	0.9 (0.8–1.0)	0.9 (0.7–1.0)	0.322
eGFR, ml/min/1.73 m^2^	79.8 (63–85)	79.8 (63.1–83.4)	83 (62.7–106.5)	0.125
**Serum LDH**^@^, U/L	643 (481–784.3)	631 (492–796.5)	656 (419.5–752.5)	0.499
**Uric acid profiles**				
SUA, mg/dl	8 (6-9)	8.9 (8-9)	5 (4.6–6)	< 0.001
24–hr UUA, mg	983 (612.3–1,096)	1037 (718.5–1,122)	741.4 (483–1,074)	0.046
Hyperuricosuria^$^, *n* (%)	51 (68.9)	40 (75.5)	11 (52.4)	0.027

As the continuous data in this study did not follow a normal distribution, all data were reported as medians with interquartile ranges (IQR).

BMI, body mass index; MPN, myeloproliferative neoplasm; CML, chronic myeloid leukemia; ET, essential thrombocytosis; PV, polycythemia vera; WBC, white blood cells count; eGFR, estimated glomerular filtration rate; LDH, lactate dehydrogenase; SUA, serum uric acid; 24-h UUA, 24-h urine uric acid.

^*^Serum uric acid > 6.8 mg/dl, ^@^Normal range of serum LDH: 150–245 U/L, ^$^24-h UUA > 700 mg.

**Figure 2:**
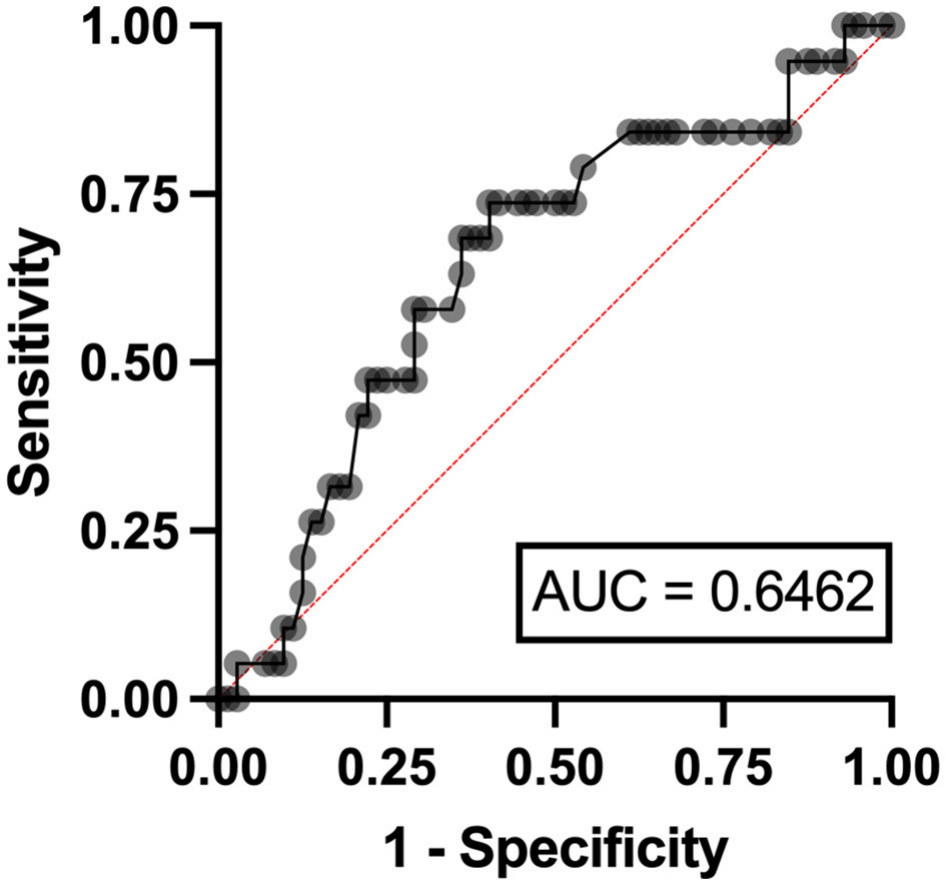
Area under a receiver operating characteristic (AuROC) curve for eGFR and hyperuricemia in patients with lymphoma.

**Figure 3:**
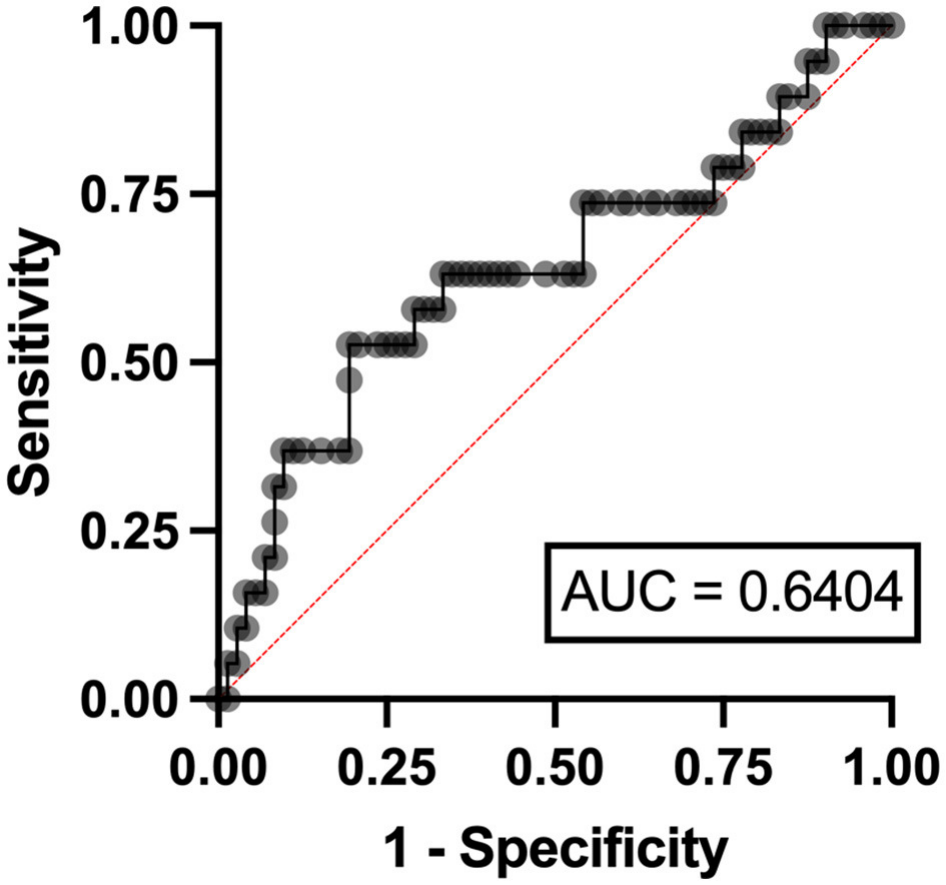
Area under a receiver operating characteristic (AuROC) curve for serum LDH and hyperuricemia in patients with lymphoma.

In the multivariable analysis, independent risk factors associated with hyperuricemia in patients with lymphoma were eGFR < 90 ml/min/1.73 m^2^ (odds ratio (OR) 3.24, 95% confidence interval (CI) 1.95–11.07, *p* = 0.006) with an area under a receiver operating characteristics (AuROC) curve of 0.6462 (Figure 2) and serum LDH ≥ 250 mg/dl (OR 2.07, 95%CI 1.62-6.97, *p* = 0.039) with AuROC curve of 0.6404 (Figure 3). Hyperuricosuria was related to hyperuricemia in both lymphoma and MPN patients (Table 4, Supplementary Table S1).

**Table 4:** Univariable and multivariable analysis for patients with hyperuricemia.

**Disease**	**Covariates**	**Values**	**Hyperuricemia (n/total)**	**Univariable analysis**		**Multivariable analysis**	
				**OR (95%CI)**	***P*** **-value**	**OR (95%CI)**	***P*** **-value**
Lymphoma	eGFR	≥90 ml/min/1.73 m^2^	5/91	Reference			
		< 90 ml/min/1.73 m^2^	14/91	1.79 (1.08–6.98)	0.035	3.24 (1.95–11.07)	0.006
	Serum LDH	< 250 U/L	7/91	Reference			
		≥250 U/L	12/91	1.10 (1.01–1.20)	0.009	2.07 (1.62–6.97)	0.039
	24-h UUA	≤ 700 mg	8/91	Reference			
		>700 mg	11/91	3.6 (7.26–17.76)	< 0.001	4.97 (7.12–17.64)	< 0.001
MPN	Sex	Female	21/74	Reference			
		Male	32/74	1.29 (0.95–1.76)	0.087	3.54 (0.91–13.79)	0.068
	24-h UUA	≤ 700 mg	13/74	Reference			
		>700 mg	40/74	2.79 (1.79–8.08)	0.027	2.96 (1.92–7.94)	0.037

MPN, myeloproliferative neoplasm; eGFR, estimated glomerular filtration rate; LDH, lactate dehydrogenase; 24-h UUA, 24-h urine uric acid.

Regarding hyperuricosuria, lymphoma patients with hyperuricosuria had a median 24-h UUA of 887.3 mg (772.8–1,049) compared to 389.6 mg (243.2–480.2) in patients with normouricosuria. Lymphoma patients with hyperuricosuria were younger (median age of 51.5 years vs. 61 years, *p* = 0.021), males predominant (85.7% vs. 42.9%, p = 0.009) with lower platelet count (median platelet 145 x 10^9^ vs. 251 x 10^9^, *p* = 0.019). Median serum LDH was higher in hyperuricosuria patients (597 U/L vs. 249 U/L, *p* = 0.013). The proportion of patients with hyperuricemia was higher in patients with hyperuricosuria than in the normouricosuria group (78.6% vs. 10.4%, *p* < 0.001) (Table 5).

**Table 5:** Characteristics of lymphoma patients separated by hyperuricosuria.

**Characteristics**	**All patients**	Hyperuricosuria^*^	**Normouricosuria**	***P*-value**
	**(*****n*** = **91)**	**(*****n*** = **14)**	**(*****n*** = **77)**	
**Age**, years	60 (51–69)	51.5 (39–61)	61 (54–70)	0.021
≥60 years, *n* (%)	46 (50.5)	4 (28.6)	42 (54.5)	0.083
**Male**, n (%)	45 (49.5)	12 (85.7)	33 (42.9)	0.009
**BMI**, kg/m^2^	21.1 (19–22.7)	22.4 (21–22.9)	20.5 (18.8–22.6)	0.173
**Comorbidities**, ***n*** **(%)**				
Diabetes mellitus	6 (6.6)	1 (7.2)	5 (6.5)	0.928
Hypertension	18 (19.8)	2 (14.3)	16 (20.8)	0.225
**Lymphoma subtype**, ***n*** **(%)**				
DLBCL	77 (84.6)	12 (85.7)	65 (84.4)	0.633
Indolent lymphoma^§^	14 (15.4)	2 (14.3)	12 (15.6)	0.713
**Ann Arbor staging**, ***n*** **(%)**				
Stage I–II	29 (31.9)	4 (28.6)	25 (32.5)	0.453
Stage III–IV	62 (68.1)	10 (71.4)	52 (67.5)	0.375
**IPI risk group**, ***n*** **(%)**				
Low (0-1)	25 (27.5)	3 (21.4)	22 (28.6)	0.288
Low-intermediate (2)	14 (15.4)	1 (7.2)	13 (16.8)	0.142
High-intermediate (3)	22 (24.2)	5 (35.7)	17 (22.1)	0.228
High (4-5)	30 (32.9)	5 (35.7)	25 (32.5)	0.482
**Bulky disease**^#^, *n* (%)	20 (21.9)	3 (21.4)	17 (22.1)	0.552
**Laboratory parameters**				
**Complete blood count**				
Hemoglobin, g/dl	10.6 (8.9–12.6)	10 (8.1–12.6)	11.1 (9–12.6)	0.507
WBC, x10^9^/L	6.7 (4.6–8.7)	7.5 (4.6–10.3)	6.6 (4.9–8.6)	0.061
Platelet, x10^9^/L	243 (146–326.4)	145 (56–244)	251 (167–336)	0.019
**Renal function**				
serum creatinine, mg/dl	0.8 (0.6–0.9)	0.9 (0.7–1.1)	0.8 (0.7–0.9)	0.440
eGFR, ml/min/1.73 m^2^	89.0 (73.0–103.3)	81.5 (73–114)	90 (75.7–102)	0.790
**Serum LDH**, U/L	254 (210–559)	597 (254–661)	249 (210–421)	0.013
≥250, n (%)	49 (53.8)	11 (78.6)	38 (49.4)	0.025
**Uric profiles**				
SUA, mg/dl	5.3 (3.5–6.6)	5.5 (3.7–7.6)	5.3 (3.5–6.6)	0.485
Hyperuricemia^$^, n (%)	19 (20.9)	11 (78.6)	8 (10.4)	< 0.001
24-hr UUA, mg	420 (277–561.5)	887.3 (772.8–1,049)	389.6 (243.2–480.2)	< 0.001

As the continuous data in this study did not follow a normal distribution, all data were reported as medians with interquartile ranges (IQR).

BMI, body mass index; DLBCL, diffuse large B-cell lymphoma; IPI, International Prognostic Index; WBC, white blood cells count; eGFR, estimated glomerular filtration rate; LDH, lactate dehydrogenase; SUA, serum uric acid; 24-h UUA, 24-h urine uric acid.

^§^Indolent lymphoma: marginal zone lymphoma (n = 9) and follicular lymphoma (n = 5), ^*^24-h UUA > 700 mg, ^#^Bulky disease: tumor diameter > 7.5 cm, ^$^Serum uric acid > 6.8 mg/dl.

Among MPN patients, those with hyperuricosuria had lower hemoglobin level (median hemoglobin level 9.4 g/dl vs. 12 g/dl, *p* = 0.006), higher white blood cells count (median WBC count 186.5 × 10^9^/L vs. 18.5 × 10^9^/L, *p* = 0.004), and lower platelet count (median platelet 521.9 × 10^9^/L vs. 655 × 10^9^/L, *p* = 0.017) compared to those with normouricosuria. For the MPN subtype, CML patients had a higher proportion of hyperuricosuria (78.4% vs. 39.1%, *p* = 0.002), while the ET patients had a lower proportion of patients with hyperuricosuria (9.8% vs. 34.8%, *p* = 0.013). MPN patients with hyperuricosuria had lower median eGFR (*p* = 0.034) but higher median serum LDH (702 U/L vs. 491 U/L, *p* = 0.003). Hyperuricemia was higher in patients with hyperuricosuria than in the normouricosuria group (78.7% vs. 56.5%, *p* = 0.037) (Table 6).

**Table 6:** Characteristics of myeloproliferative neoplasm patients separated by hyperuricosuria.

**Characteristics**	**All patients**	Hyperuricosuria^*^	**Normouricosuria**	***P*-value**
	**(*****n*** = **74)**	**(*****n*** = **51)**	**(*****n*** = **23)**	
**Age**, years	48 (39.7–58)	48 (39–56)	49 (46–59)	0.472
**Male**, *n* (%)	40 (54.1)	29 (56.9)	11 (47.8)	0.615
**BMI**, kg/m^2^	21.5 (19-23)	21.6 (19-23)	21 (19.2–24)	0.842
**Comorbidities**, ***n*** **(%)**				
Diabetes mellitus	2 (2.7)	1 (1.9)	1 (4.3)	0.472
Hypertension	6 (8.1)	5 (9.8)	1 (4.3)	0.659
**MPN subtype**, ***n*** **(%)**				
CML	49 (66.2)	40 (78.4)	9 (39.1)	0.002
ET	13 (17.6)	5 (9.8)	8 (34.8)	0.013
PV	12 (16.2)	6 (11.8)	6 (26.1)	0.173
**Laboratory parameters**				
**Complete blood count**				
Hemoglobin, g/dl	10.2 (8.5–12.3)	9.4 (8.4–11.1)	12 (9.6–18.5)	0.006
WBC, × 10^9^/L	94.1 (18.6–263.6)	186.5 (55.7–274.5)	18.5 (9.4–65.2)	0.004
Platelet, × 10^9^/L	545.9 (364.5–677.7)	521.9 (334.3–635.2)	655 (413.8–875.6)	0.017
**Renal function**				
Serum creatinine, mg/dl	0.9 (0.7–1.0)	0.9 (0.8–1.0)	0.9 (0.7–1.0)	0.489
eGFR, ml/min/1.73 m^2^	79.8 (63–85)	79.8 (62.3–83.6)	80.4 (77.9–99)	0.022
**Serum LDH**, U/L	643 (481–784.3)	702 (556–801)	491 (298–563)	0.003
≥640, n (%)	37 (50)	32 (62.7)	5 (21.7)	0.002
**Uric profiles**				
SUA, mg/dl	8 (6-9)	8 (7.2–9)	7.2 (4.6–9)	0.025
Hyperuricemia^$^, n (%)	53 (71.6)	40 (78.7)	13 (56.5)	0.037
24-h UUA, mg	983 (612.3–1,096)	1072 (972–1,201)	516 (402–601)	< 0.001

As the continuous data in this study did not follow a normal distribution, all data were reported as medians with interquartile ranges (IQR).

BMI, body mass index; MPN, myeloproliferative neoplasm; CML, chronic myeloid leukemia; ET, essential thrombocytosis; PV, polycythemia vera; WBC, white blood cells count; eGFR, estimated glomerular filtration rate; LDH, lactate dehydrogenase; SUA, serum uric acid.

^*^24-h UUA > 700 mg/dl, ^$^Serum uric acid > 6.8 mg/dl.

**Figure 4:**
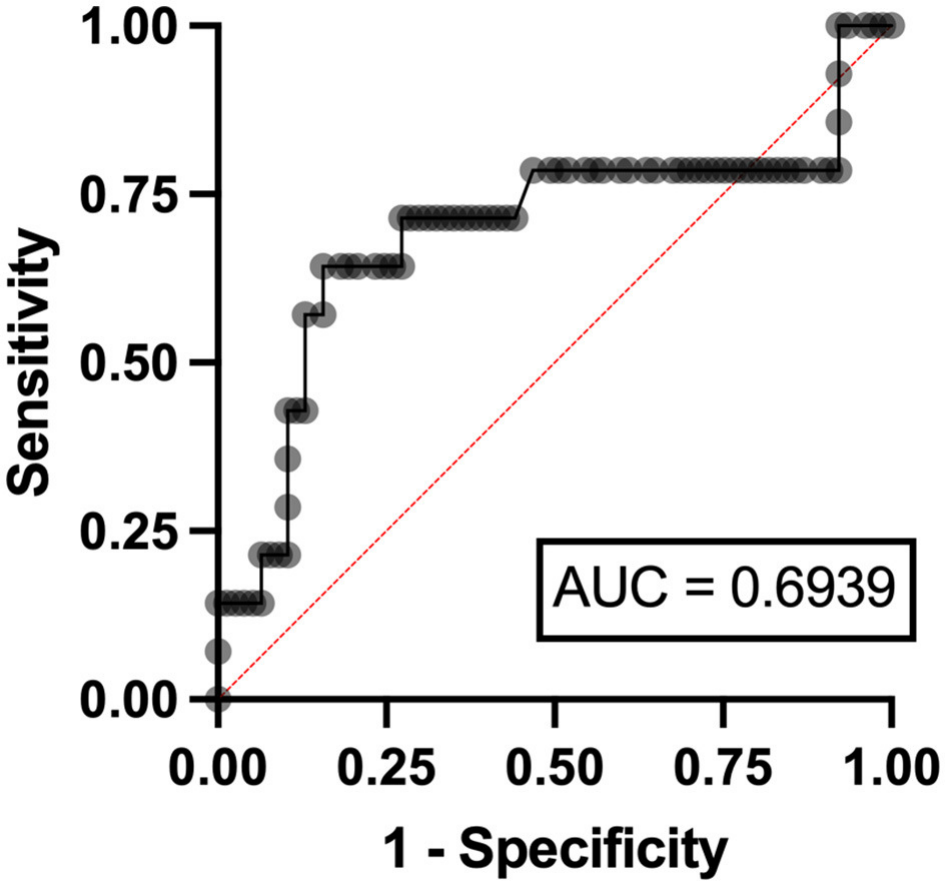
Area under a receiver operating characteristic (AuROC) curve for serum LDH and hyperuricosuria in patients with lymphoma.

**Figure 5:**
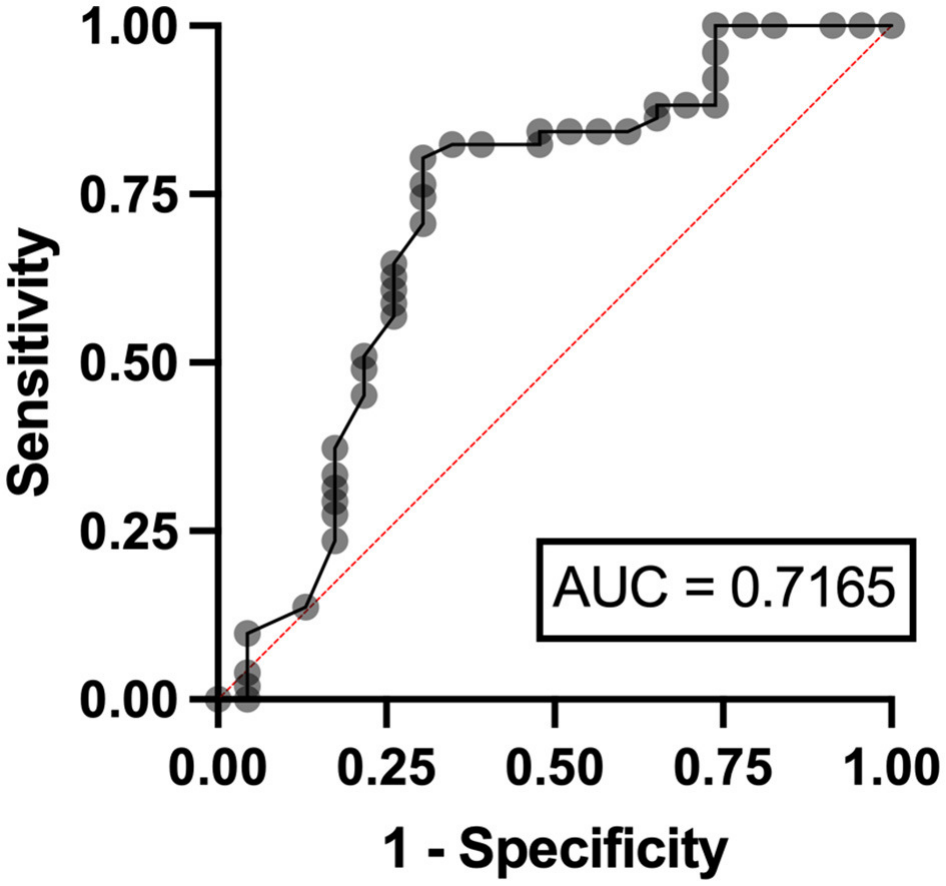
Area under a receiver operating characteristic (AuROC) curve for hemoglobin level and hyperuricosuria in patients with MPN.

**Figure 6:**
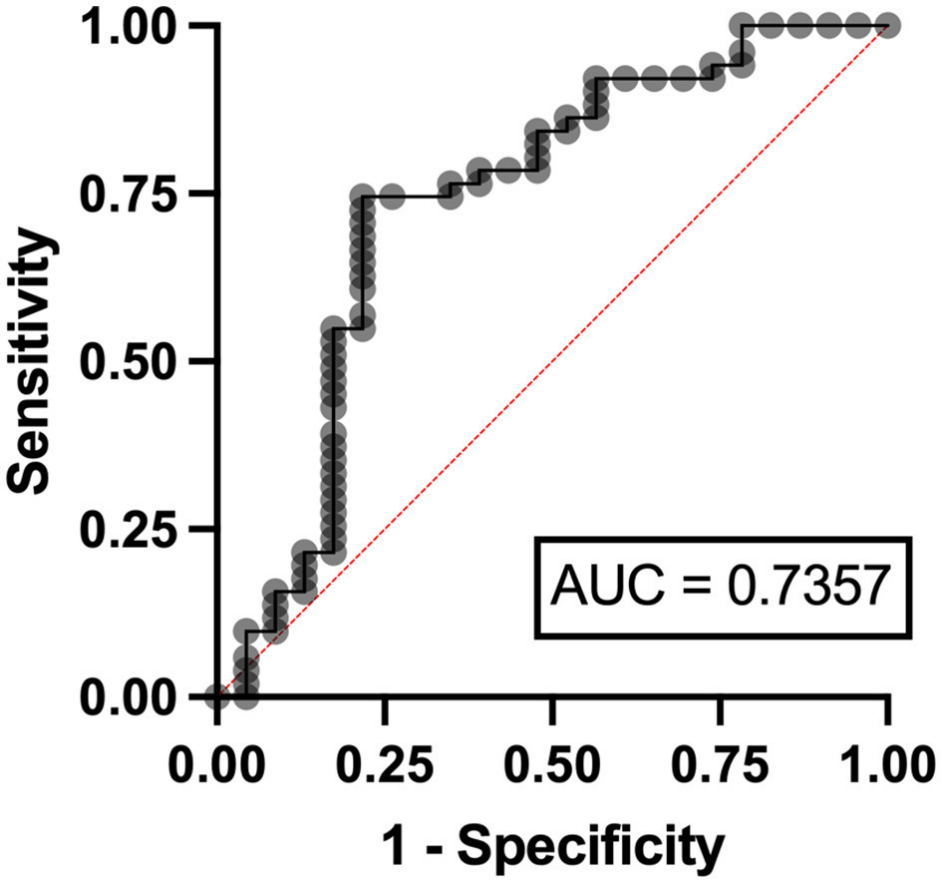
Area under a receiver operating characteristic (AuROC) curve for serum LDH and hyperuricosuria in patients with MPN.

Independent risk factors for hyperuricosuria in lymphoma patients were male sex (OR 9.11, 95%CI 1.12–7.78, *p* = 0.038) and LDH ≥ 250 mg/dl (OR 2.37, 95%CI 1.56–14.29, *p* = 0.036) with AuROC curve of 0.6939 (Figure 4). However, hemoglobin level < 10 g/dl and serum LDH ≥ 640 mg/dl were independent risk factors for hyperuricosuria in MPN patients (OR 1.88, 95%CI 1.42–8.39, *p* = 0.045 with AuROC curve of 0.7165 and OR 6.21, 95%CI 1.49–25.74, *p* = 0.012 with AuROC curve of 0.7357) (Figures 5, 6). Hyperuricemia was the related factor hyperuricosuria in both MPN and lymphoma patients (Table 7, Supplementary Table S2).

**Table 7:** Univariable and multivariable analysis for patients with hyperuricosuria.

**Disease**	**Covariates**	**Values**	**Hyperuricosuria**	**Univariable analysis**		**Multivariable analysis**	
			**(n/total)**	**OR (95%CI)**	***P*** **-value**	**OR (95%CI)**	***P*** **-value**
Lymphoma	Age	< 60 years	10/91	Reference			
		≥60 years	4/91	0.12 (0.02–0.94)	0.083	0.57 (0.09–3.36)	0.536
	Sex	Female	2/91	Reference			
		Male	12/91	8.18 (1.07–6.76)	0.009	9.11 (1.12–7.78)	0.038
	Serum LDH	< 250 U/L	3/91	Reference			
		≥250 U/L	11/91	3.76 (1.72–14.56)	0.025	2.37 (1.56–14.29)	0.036
	SUA	≤ 6.8 mg/dL	3/91	Reference			
		>6.8 mg/dL	11/91	3.6 (7.25–17.76)	< 0.001	2.9 (5.78–17.89)	< 0.001
MPN	Hemoglobin	≥10 g/dl	21//74	Reference			
		< 10 g/dl	30/74	4.05 (1.37–11.98)	0.012	1.88 (1.42–8.39)	0.045
	WBC	< 94 × 10^9^/L	18/74	Reference			
		≥94 × 10^9^/L	33/74	8.71 (2.57–29.54)	0.001	5.31 (0.81–35.25)	0.084
	Platelet count	< 545 × 10^9^/L	30/74	Reference			
		≥545 × 10^9^/L	21/74	0.31 (0.11–0.87)	0.027	0.99 (0.99–1.12)	0.159
	CML	No	11/74	Reference			
		Yes	40/74	5.66 (1.94–16.51)	0.002	2.32 (0.14–37.44)	0.553
	ET	No	46/74	Reference			
		Yes	5/74	0.21 (0.06–0.72)	0.013	0.39 (0.05–3.10)	0.376
	Serum LDH	< 640 U/L	19/74	Reference			
		≥640 U/L	32/74	6.06 (1.94–18.99)	0.002	6.21 (1.49–25.74)	0.012
	SUA	≤ 6.8 mg/dl	11/74	Reference			
		> 6.8 mg/dl	40/74	2.79 (1.97–8.08)	0.037	6.47 (1.22–14.39)	0.039

LDH, lactate dehydrogenase; SUA, serum uric acid; WBC, white blood cell count; CML, chronic myeloid leukemia; ET, essential thrombocytosis; MPN, myeloproliferative neoplasm.

## 4 Discussion

This study found a 43.6% overall prevalence of hyperuricemia in hematologic malignancies, almost 3.5 times more common in MPN than in lymphoma patients. The independent risk factors for developing hyperuricemia in lymphoma patients were impaired renal function and elevated serum LDH. Moreover, hyperuricosuria was found in 39.4% of patients, almost 4.5 times more common in MPN than lymphoma patients. The independent risk factor of hyperuricosuria was anemia in lymphoma and elevated serum LDH in both of lymphoma and MPN patients.

The prevalence of hyperuricemia in this study was consistent with the previous reports (1, 3, 4, 14–16). One small-scale study enrolled 12 lymphoma patients with a 58.3% prevalence of hyperuricemia, and 41.6% had hyperuricosuria (14). Another study of uric acid metabolism, involving 186 patients with hematologic malignancies, including leukemia, MPN and multiple myeloma reported an overall prevalence of hyperuricemia of 65.5%. Notably, two-thirds of the patients with hyperuricemia in this study had MPN and CML, and there were no patients with lymphoma included in the study (15). A retrospective study from Japan included 418 hematologic malignancies patients (lymphoma in almost half of cases and leukemia in one-third) found the overall prevalence of hyperuricemia of 27.8% (23.6% in lymphoma, and 45.8% in CML), which showed a similar trend to our study. Hyperuricosuria was found in only 10%, all of whom were male patients with lymphoma and acute leukemia (16). Regarding hyperuricemia and tumor lysis syndrome in patients with acute leukemia and non-Hodgkin lymphoma, hyperuricemia ranged from 12.6% to 26.5%, with the development of tumor lysis syndrome occurring in about 15.9%−27.8% of cases (1, 3). In patients with MPN, specifically ET and PV, a previous study reported a hyperuricemia prevalence of 31% and demonstrated inferior thrombosis-free survival (TFS) in patients with SUA levels above 5.06 mg/dL (4). The prevalence of hyperuricemia in MPN patients from this study differed from our study, with a hyperuricemia of 71.6%. This variation may be attributed to the slightly different SUA cutoff defining hyperuricemia and the inclusion of patients with CML, which accounted for 66.2% of MPN cases in our current study. The higher prevalence of hyperuricemia in MPN in our study may stem from the elevated white blood cell count seen in CML, reflecting a higher tumor burden and uric acid load. This study found that lower hemoglobin levels and high LDH were independent risk factors for hyperuricosuria. The exploratory hypothesis was supported by an increase in cell turnover, ineffective erythropoiesis, and reduced renal clearance, which contributed to the accumulation of uric acid and increased excretion of uric acid in the urine.

For lymphoma patients, hyperuricosuria was observed in only 15.4%. Our findings demonstrated that two-thirds of MPN patients had hyperuricosuria, with the majority of them having CML. Therefore, MPN patients with anemia and high serum LDH who had hyperuricemia should be considered the uric acid lowering therapy to prevent further complications arising from elevated serum uric acid levels.

There were several limitations in this study. As shown in this study, a separate analysis of prevalence and associated risk factors on uric profiling was performed because of the difference in the natural course of the disease and clinical characteristics between lymphoma and MPN. Therefore, it may raise the issue of an underpowered study from the small sample size in each group in terms of associated risk factors analysis. Patients with acute leukemia were also excluded because our study group prioritized emergency treatment for patient safety, particularly concerning tumor lysis syndrome. Typically, allopurinol is administered before commencing treatment in such cases. Furthermore, a single center with a cross-sectional study design may limit the generalizability of the findings. Then, the further longitudinally prospective study with the collaboration of multicenter on hematologic malignancy patients, including a matched-control group, with comprehensive monitoring of serum uric, urate excretion, and clinical correlation, would be warranted to make causal inferences. Additionally, there was a lack of data on the potential genetic alterations of a group of genes related to the net balance of renal urate absorption and secretion that might influent SUA and UUA (25). However, the strength of our study compared to all the previous studies was the analytically addressed risk factors of hyperuricemia and hyperuricosuria. The various conditions and medications that could alter SUA and UUA excretion were excluded to ensure that SUA and UUA were not interfered with by other factors, which were not mentioned in many previous studies. Also, those patients who were found to have eGFR < 60 ml/min/1.73 m^2^ after enrollment were excluded to ensure that those who participated in this study did not have impaired renal function that could interfere with SUA and UUA excretion.

## 5 Conclusion

This study found that almost half of the patients with hematologic malignancies (predominant with lymphoma and MPN) had hyperuricemia, and only one-third had hyperuricosuria (the majority being MPN and CML). Lymphoma patients with lower eGFR and high serum LDH were at risk of hyperuricemia. Elevated LDH was related to hyperuricosuria in both lymphoma and MPN patients. These findings may have implications for managing and treating hyperuricemia in patients with hematologic malignancies.

## Data availability statement

The original contributions presented in the study are included in the article/Supplementary material, further inquiries can be directed to the corresponding author.

## Ethics statement

The studies involving humans were approved by Research Ethics Committee 4, Faculty of Medicine, Chiang Mai University. The studies were conducted in accordance with the local legislation and institutional requirements. The participants provided their written informed consent to participate in this study.

## Author contributions

TK: Conceptualization, Data curation, Formal analysis, Investigation, Methodology, Validation, Writing—review & editing. TR: Conceptualization, Data curation, Formal analysis, Investigation, Methodology, Writing—original draft, Writing—review & editing. TP: Writing—review & editing. NH: Writing—review & editing. PP: Writing—review & editing. SH: Writing—review & editing. CC-A: Writing—review & editing. ER: Writing—review & editing. AT: Writing—review & editing. LN: Writing—review & editing. WL: Conceptualization, Methodology, Writing—review & editing.

## References

[1] Annemans L Moeremans K Lamotte M Garcia Conde J van den Berg H Myint H. Incidence, medical resource utilisation and costs of hyperuricemia and tumour lysis syndrome in patients with acute leukaemia and non-Hodgkin's lymphoma in four European countries. Leuk Lymphoma. (2003) 44:77–83. 10.1080/1042819021000054661 12691145

[2] Tsimberidou AM Keating MJ. Hyperuricemic syndromes in cancer patients. Contrib Nephrol. (2005) 147:47–60. 10.1159/000082541 15604605

[3] Sevinir B Demirkaya M Baytan B Gunes AM. Hyperuricemia and tumor lysis syndrome in children with non-Hodgkin's lymphoma and acute lymphoblastic leukemia. Turk J Haematol. (2011) 28:52–9. 10.5152/tjh.2011.06 27263942

[4] Krecak I Lucijanic M Gveric-Krecak V Durakovic N. Hyperuricemia might promote thrombosis in essential thrombocythemia and polycythemia vera. Leuk Lymphoma. (2020) 61:1744–7. 10.1080/10428194.2020.1731503 32096431

[5] Li C Hsieh MC Chang SJ. Metabolic syndrome, diabetes, and hyperuricemia. Curr Opin Rheumatol. (2013) 25:210–6. 10.1097/BOR.0b013e32835d951e 23370374

[6] Mortada I. Hyperuricemia, type 2 diabetes mellitus, and hypertension: an emerging association. Curr Hypertens Rep. (2017) 19:69. 10.1007/s11906-017-0770-x 28770533

[7] Zhang S Wang Y Cheng J Huangfu N Zhao R Xu Z. Hyperuricemia and cardiovascular disease. Curr Pharm Des. (2019) 25:700–9. 10.2174/1381612825666190408122557 30961478

[8] Gaubert M Bardin T Cohen-Solal A Diévart F Fauvel J-P Guieu R. Hyperuricemia and hypertension, coronary artery disease, kidney disease: from concept to practice. Int J Mol Sci. (2020) 21:4066. 10.3390/ijms21114066 33561034 PMC7312288

[9] Copur S Demiray A Kanbay M. Uric acid in metabolic syndrome: does uric acid have a definitive role? Eur J Intern Med. (2022) 103:4–12. 10.1016/j.ejim.2022.04.022 35508444

[10] Piani F Agnoletti D Borghi C. Advances in pharmacotherapies for hyperuricemia. Expert Opin Pharmacother. (2023) 24:737–45. 10.1080/14656566.2023.2197591 36999206

[11] Mirheydar HS Banapour P Massoudi R Palazzi KL Jabaji R Reid EG. What is the incidence of kidney stones after chemotherapy in patients with lymphoproliferative or myeloproliferative disorders? Int Braz J Urol. (2014) 40:772–80. 10.1590/S1677-5538.IBJU.2014.06.08 25615245

[12] Song L Maalouf NM. Nephrolithiasis. In: Feingold KR Anawalt B Blackman MR et al., editors. Endotext. South Dartmouth, MA. (2000).

[13] Wiederkehr MR Moe OW. Uric acid nephrolithiasis: a systemic metabolic disorder. Clin Rev Bone Miner Metab. (2011) 9:207–17. 10.1007/s12018-011-9106-6 25045326 PMC4100778

[14] Primikirios N Stutzman L Sandberg AA. Uric acid excretion in patients with malignant lymphomas. Blood. (1961) 17:701–18. 10.1182/blood.V17.6.701.701 13738036

[15] Lynch EC. Uric acid metabolism in proliferative diseases of the marrow. Arch Intern Med. (1962) 109:639–53. 10.1001/archinte.1962.03620180001001 14467574

[16] Oka Y Tashiro H Sirasaki R Yamamoto T Akiyama N Kawasugi K. Hyperuricemia in hematologic malignancies is caused by an insufficient urinary excretion. Nucleosides Nucleotides Nucleic Acids. (2014) 33:434–8. 10.1080/15257770.2013.872274 24940701

[17] Denman M Szur L Ansell BM. Hyperuricaemia in polycythaemia vera. Ann Rheum Dis. (1966) 25:340–4. 10.1136/ard.25.4.340 5947579 PMC2453349

[18] Schlesinger N Norquist JM Watson DJ. Serum urate during acute gout. J Rheumatol. (2009) 36:1287–9. 10.3899/jrheum.080938 19369457

[19] Neogi T. Clinical practice. Gout N Engl J Med. (2011) 364:443–52. 10.1056/NEJMcp1001124 21288096

[20] Emmerson BT. The management of gout. N Engl J Med. (1996) 334:445–51. 10.1056/NEJM199602153340707 8552148

[21] Perez-Ruiz F Calabozo M Erauskin GG Ruibal A Herrero-Beites AM. Renal underexcretion of uric acid is present in patients with apparent high urinary uric acid output. Arthritis Rheum. (2002) 47:610–3. 10.1002/art.10792 12522834

[22] Yang CY Chen FA Chen CF Liu WS Shih CJ Ou SM. Diagnostic accuracy of urine protein/creatinine ratio is influenced by urine concentration. PLoS ONE. (2015) 10:e0137460. 10.1371/journal.pone.0137460 26353117 PMC4564100

[23] Stevens PE Levin A Kidney Disease: Improving Global Outcomes Chronic Kidney Disease Guideline Development Work Group M. Evaluation and management of chronic kidney disease: synopsis of the kidney disease: improving global outcomes 2012 clinical practice guideline. Ann Intern Med. (2013) 158:825–30. 10.7326/0003-4819-158-11-201306040-00007 23732715

[24] Naing L Nordin RB Abdul Rahman H Naing YT. Sample size calculation for prevalence studies using Scalex and ScalaR calculators. BMC Med Res Methodol. (2022) 22:209. 10.1186/s12874-022-01694-7 35907796 PMC9338613

[25] Reginato AM Mount DB Yang I Choi HK. The genetics of hyperuricaemia and gout. Nat Rev Rheumatol. (2012) 8:610–21. 10.1038/nrrheum.2012.144 22945592 PMC3645862

