# Supplementary material for: Prevalence and risk factors for hyperuricemia and hyperuricosuria in patients with hematologic malignancies
Source: Front Med (Lausanne). 2024 May 30;11:1343000. doi: 10.3389/fmed.2024.1343000 (PMC11169800; doi:10.3389/fmed.2024.1343000)
Supplement: Supplementary file 1 [file Data_Sheet_1.docx]

**Table S1.** **Univariable with all parameters and multivariable analysis for patients with hyperuricemia**

| **Disease** | **Covariates** | **Values** | **Hyperuricemia** | **Univariable analysis** | | **Multivariable analysis** | | |  |
| --- | --- | --- | --- | --- | --- | --- | --- | --- | --- |
|  |  |  | **(n/total)** | **OR (95%CI)** | ***P*-value** | | **OR (95%CI)** | ***P*-value** | |
| **Lymphoma** | Age | < 60 years | 0/91 | Reference |  | |  |  | |
|  |  | ≥ 60 years | 10/91 | 0.96 (0.93-1.01) | 0.131 | | - | - | |
|  | Sex | Female | 10/91 | Reference |  | |  |  | |
|  |  | Male | 9/91 | 0.45 (0.14-1.41) | 0.168 | | - | - | |
|  | BMI | < 21 kg/m^2^ | 10/91 | Reference |  | |  |  | |
|  |  | ≥ 21 kg/m^2^ | 9/91 | 1.17 (0.96-1.43) | 0.121 | | - | - | |
|  | Diabetes mellitus | No | 18/91 | Reference |  | |  |  | |
|  |  | Yes | 1/91 | 0.86 (0.91-3.53) | 0.520 | | - | - | |
|  | Hypertension | No | 16/91 | Reference |  | |  |  | |
|  |  | Yes | 3/91 | 0.53 (0.08-3.31) | 0.495 | | - | - | |
|  | Lymphoma type | Indolent | 1/91 | Reference |  | |  |  | |
|  |  | DLBCL | 18/91 | 2.27 (0.57-9.12) | 0.250 | | - | - | |
|  | Staging | Stage I-II | 6/91 | Reference |  | |  |  | |
|  |  | Stage III-IV | 13/91 | 1.82 (0.62-3.45) | 0.423 | | - | - | |
|  | IPI risk group | Low to Low-int | 7/91 | Reference |  | |  |  | |
|  |  | High-int to High | 12/91 | 2.12 (0.78-2.32) | 0.392 | | - | - | |
|  | Bulky disease | No | 14/91 | Reference |  | |  |  | |
|  |  | Yes | 5/91 | 1.29 (0.88-4.52) | 0.62 | | - | - | |
|  | Hemoglobin | < 10 g/dl | 7/91 | Reference |  | |  |  | |
|  |  | ≥ 10 g/dl | 12/91 | 1.14 (0.91-1.46) | 0.264 | | - | - | |
|  | WBC | < 6.7 x 10^9^/L | 9/91 | Reference |  | |  |  | |
|  |  | ≥ 6.7 x 10^9^/L | 10/91 | 1.01 (0.99-1.01) | 0.297 | | - | - | |
|  | Platelet | <243 x 10^9^/L | 7/91 | Reference |  | |  |  | |
|  |  | ≥ 243 x 10^9^/L | 12/91 | 0.99 (0.99-1.02) | 0.524 | | - | - | |
|  | Serum creatinine | < 0.8 mg/dl | 4/91 | Reference |  | |  |  | |
|  |  | ≥ 0.8 mg/dl | 15/91 | 6.22 (0.64-60.55) | 0.115 | | - | - | |
|  | eGFR | ≥ 90 ml/min/1.73 m^2^ | 5/91 | Reference |  | |  |  | |
|  |  | < 90 ml/min/1.73 m^2^ | 14/91 | 1.79 (1.08-6.98) | 0.035 | | 3.24 (1.95-11.07) | 0.006 | |
|  | Serum LDH | < 250 U/L | 7/91 | Reference |  | |  |  | |
|  |  | ≥ 250 U/L | 12/91 | 1.10 (1.01-1.20) | 0.009 | | 2.07 (1.62-6.97) | 0.039 | |
|  | 24-hr UUA | ≤ 700 mg | 8/91 | Reference |  | |  |  | |
|  |  | > 700 mg | 11/91 | 3.6 (7.26-17.76) | <0.001 | | 4.97 (7.12-17.64) | <0.001 | |
| **MPN** | Age | < 48 years | 40/74 | Reference |  | |  |  | |
|  |  | ≥ 48 years | 13/74 | 1.03 (0.96-1.10) | 0.399 | | - | - | |
|  | Sex | Female | 21/74 | Reference |  | |  |  | |
|  |  | Male | 32/74 | 1.29 (0.95-1.76) | 0.087 | | 3.54 (0.91-13.79) | 0.068 | |
|  | BMI | < 21.5 kg/m^2^ | 25/74 | Reference |  | |  |  | |
|  |  | ≥ 21.5 kg/m^2^ | 28/74 | 1.25 (0.93-1.69) | 0.130 | | - | - | |
|  | Diabetes mellitus | No | 52/74 | Reference |  | |  |  | |
|  |  | Yes | 1/74 | 0.18 (0.14-6.87) | 0.355 | | - | - | |
|  | Hypertension | No | 48/74 | Reference |  | |  |  | |
|  |  | Yes | 5/74 | 1.56 (0.06-39.38) | 0.786 | | - | - | |
|  | CML | No | 18/74 | Reference |  | |  |  | |
|  |  | Yes | 35/74 | 2.01 (0.39-10.31) | 0.407 | | - | - | |
|  | ET | No | 45/74 | Reference |  | |  |  | |
|  |  | Yes | 8/74 | 1.38 (0.48-4.23) | 0.389 | | - | - | |
|  | PV | No | 43/74 | Reference |  | |  |  | |
|  |  | Yes | 10/74 | 0.64 (0.18-2.29) | 0.494 | | - | - | |
|  | Hemoglobin | < 10 g/dl | 28/74 | Reference |  | |  |  | |
|  |  | ≥ 10 g/dl | 25/74 | 0.59 (0.35-1.01) | 0.189 | | - | - | |
|  | WBC | < 94 x 10^9^/L | 28/74 | Reference |  | |  |  | |
|  |  | ≥ 94 x 10^9^/L | 25/74 | 0.99 (0.99-1.10) | 0.683 | | - | - | |
|  | Platelet | <545 x 10^9^/L | 29/74 | Reference |  | |  |  | |
|  |  | ≥ 545 x 10^9^/L | 24/74 | 0.89 (0.99-1.01) | 0.462 | | - | - | |
|  | Serum creatinine | < 0.9 mg/dl | 20/74 | Reference |  | |  |  | |
|  |  | ≥ 0.9 mg/dl | 33/74 | 4.68 (0.43-13.9) | 0.310 | | - | - | |
|  | eGFR | ≥ 80 ml/min/1.73 m^2^ | 22/74 | Reference |  | |  |  | |
|  |  | < 80 ml/min/1.73 m^2^ | 31/74 | 0.92 (0.95-1.34) | 0.737 | | - | - | |
|  | Serum LDH | < 640 U/L | 27/74 | Reference |  | |  |  | |
|  |  | ≥ 640 U/L | 26/74 | 1.01 (0.98-1.02) | 0.804 | | - | - | |
|  | 24-hr UUA | ≤ 700 mg | 13/74 | Reference |  | |  |  | |
|  |  | > 700 mg | 40/74 | 2.79 (1.79-8.08) | 0.027 | | 2.96 (1.92-7.94) | 0.037 | |

MPN: myeloproliferative neoplasm, eGFR: estimated glomerular filtration rate, LDH: lactate dehydrogenase, 24-hr UUA: 24-hour urine uric acid

**Table S2.** **Univariable with all parameters and multivariable analysis for patients with hyperuricosuria**

| **Disease** | **Covariates** | **Values** | **Hyperuricosuria** | **Univariable analysis** | | | **Multivariable analysis** | | |
| --- | --- | --- | --- | --- | --- | --- | --- | --- | --- |
|  |  |  | **(n/total)** | **OR (95%CI)** | ***P*-value** | **OR (95%CI)** | | ***P*-value** |  |
| **Lymphoma** | Age | < 60 years | 10/91 | Reference |  |  | |  |  |
|  |  | ≥ 60 years | 4/91 | 0.12 (0.02-0.94) | 0.083 | 0.57 (0.09-3.36) | | 0.536 |  |
|  | Sex | Female | 2/91 | Reference |  |  | |  |  |
|  |  | Male | 12/91 | 8.18 (1.07-6.76) | 0.009 | 9.11 (1.12-7.78) | | 0.038 |  |
|  | BMI | < 21 kg/m^2^ | 4/91 | Reference |  |  | |  |  |
|  |  | ≥ 21 kg/m^2^ | 10/91 | 1.09 (0.85-1.41) | 0.483 | - | | - |  |
|  | Diabetes mellitus | No | 13/91 | Reference |  |  | |  |  |
|  |  | Yes | 1/91 | 1.16 (0.05-29.35) | 0.926 | - | | - |  |
|  | Hypertension | No | 12/91 | Reference |  |  | |  |  |
|  |  | Yes | 2/91 | 0.41 (0.03-6.07) | 0.516 | - | | - |  |
|  | Lymphoma type | Indolent | 2/91 | Reference |  |  | |  |  |
|  |  | DLBCL | 12/91 | 1.73 (0.20-14.95) | 0.615 | - | | - |  |
|  | Staging | Stage I-II | 4/91 | Reference |  |  | |  |  |
|  |  | Stage III-IV | 10/91 |  |  | - | | - |  |
|  | IPI risk group | Low to Low-int | 4/91 | Reference |  |  | |  |  |
|  |  | High-int to High | 10/91 |  |  | - | | - |  |
|  | Bulky disease | No | 11/91 | Reference |  |  | |  |  |
|  |  | Yes | 3/91 |  |  | - | | - |  |
|  | Hemoglobin | < 10 g/dl | 7/91 | Reference |  |  | |  |  |
|  |  | ≥ 10 g/dl | 7/91 | 0.99 (0.69-1.42) | 0.995 | - | | - |  |
|  | WBC | < 6.7 x 10^9^/L | 5/91 | Reference |  |  | |  |  |
|  |  | ≥ 6.7 x 10^9^/L | 9/91 | 1.01 (0.99-1.10) | 0.271 | - | | - |  |
|  | Platelet | <243 x 10^9^/L | 10/91 | Reference |  |  | |  |  |
|  |  | ≥ 243 x 10^9^/L | 4/91 | 0.98 (0.87-1.43) | 0.640 | - | | - |  |
|  | Serum creatinine | < 0.8 mg/dl | 5/91 | Reference |  |  | |  |  |
|  |  | ≥ 0.8 mg/dl | 9/91 | 0.66 (0.40-9.41) | 0.908 | - | | - |  |
|  | eGFR | ≥ 90 ml/min/1.73 m^2^ | 6/91 | Reference |  |  | |  |  |
|  |  | < 90 ml/min/1.73 m^2^ | 8/91 | 0.99 (0.91-1.11) | 0.996 | - | | - |  |
|  | Serum LDH | < 250 U/L | 3/91 | Reference |  |  | |  |  |
|  |  | ≥ 250 U/L | 11/91 | 3.76 (1.72-14.56) | 0.025 | 2.37 (1.56-14.29) | | 0.036 |  |
|  | SUA | ≤ 6.8 mg/dL | 3/91 | Reference |  |  | |  |  |
|  |  | > 6.8 mg/dL | 11/91 | 3.6 (7.25-17.76) | <0.001 | 2.9 (5.78-17.89) | | <0.001 |  |
| **MPN** | Age | < 48 years | 22/74 | Reference |  |  | |  |  |
|  |  | ≥ 48 years | 29/74 | 0.94 (0.87-1.03) | 0.143 | - | | - |  |
|  | Sex | Female | 22/74 | Reference |  |  | |  |  |
|  |  | Male | 29/74 | 0.96 (0.19-4.78) | 0.956 | - | | - |  |
|  | BMI | < 21.5 kg/m^2^ | 25/74 | Reference |  |  | |  |  |
|  |  | ≥ 21.5 kg/m^2^ | 26/74 | 0.94 (0.70-1.25) | 0.667 | - | | - |  |
|  | Diabetes mellitus | No | 50/74 | Reference |  |  | |  |  |
|  |  | Yes | 1/74 | 2.91 (0.32-8.90) | 0.523 | - | | - |  |
|  | Hypertension | No | 46/74 | Reference |  |  | |  |  |
|  |  | Yes | 5/74 | 2.72 (0.11-6.78) | 0.542 | - | | - |  |
|  | CML | No | 11/74 | Reference |  |  | |  |  |
|  |  | Yes | 40/74 | 5.66 (1.94-16.51) | 0.002 | 2.32 (0.14-37.44) | | 0.553 |  |
|  | ET | No | 46/74 | Reference |  |  | |  |  |
|  |  | Yes | 5/74 | 0.21 (0.06-0.72) | 0.013 | 0.39 (0.05-3.10) | | 0.376 |  |
|  | PV | No | 45/74 | Reference |  |  | |  |  |
|  |  | Yes | 6/74 | 1.82 (0.19-1.39) | 0.291 | - | | - |  |
|  | Hemoglobin | ≥ 10 g/dl | 21//74 | Reference |  |  | |  |  |
|  |  | < 10 g/dl | 30/74 | 4.05 (1.37-11.98) | 0.012 | 1.88 (1.42-8.39) | | 0.045 |  |
|  | WBC | < 94 x10^9^/L | 18/74 | Reference |  |  | |  |  |
|  |  | ≥ 94 x 10^9^/L | 33/74 | 8.71 (2.57-29.54) | 0.001 | 5.31 (0.81-35.25) | | 0.084 |  |
|  | Platelet count | < 545 x 10^9^/L | 30/74 | Reference |  |  | |  |  |
|  |  | ≥ 545 x 10^9^/L | 21/74 | 0.31 (0.11-0.87) | 0.027 | 0.99 (0.99-1.12) | | 0.159 |  |
|  | Serum creatinine | < 0.9 mg/dl | 23/74 | Reference |  |  | |  |  |
|  |  | ≥ 0.9 mg/dl | 28/74 | 2.54 (0.02-3.21) | 0.705 | - | | - |  |
|  | eGFR | ≥ 80 ml/min/1.73 m^2^ | 22/74 | Reference |  |  | |  |  |
|  |  | < 80 ml/min/1.73 m^2^ | 29/74 | 0.91 (0.83-1.93) | 0.336 | - | | - |  |
|  | Serum LDH | < 640 U/L | 19/74 | Reference |  |  | |  |  |
|  |  | ≥ 640 U/L | 32/74 | 6.06 (1.94-18.99) | 0.002 | 6.21 (1.49-25.74) | | 0.012 |  |
|  | SUA | ≤ 6.8 mg/dl | 11/74 | Reference |  |  | |  |  |
|  |  | > 6.8 mg/dl | 40/74 | 2.79 (1.97-8.08) | 0.037 | 6.47 (1.22-14.39) | | 0.039 |  |

LDH: lactate dehydrogenase, SUA: serum uric acid, WBC: white blood cell count, CML: chronic myeloid leukemia, ET: essential thrombocytosis, MPN: myeloproliferative neoplasm
